# Module-based subnetwork alignments reveal novel transcriptional regulators in malaria parasite *Plasmodium falciparum*

**DOI:** 10.1186/1752-0509-6-S3-S5

**Published:** 2012-12-17

**Authors:** Hong Cai, Changjin Hong, Jianying Gu, Timothy G Lilburn, Rui Kuang, Yufeng Wang

**Affiliations:** 1 Department of Biology, University of Texas at San Antonio, San Antonio, TX 78249, USA; 2 Department of Computer Science and Engineering, University of Minnesota Twin Cities, Minneapolis, MN 55455, USA; 3 Department of Biology, College of Staten Island, City University of New York, Staten Island, NY 10314, USA; 4 Department of Bacteriology, American Type Culture Collection, Manassas, VA 20110, USA; 5 South Texas Center for Emerging Infectious Diseases, University of Texas at San Antonio, San Antonio, TX 78249, USA

## Abstract

**Background:**

Malaria causes over one million deaths annually, posing an enormous health and economic burden in endemic regions. The completion of genome sequencing of the causative agents, a group of parasites in the genus *Plasmodium*, revealed potential drug and vaccine candidates. However, genomics-driven target discovery has been significantly hampered by our limited knowledge of the cellular networks associated with parasite development and pathogenesis. In this paper, we propose an approach based on aligning neighborhood PPI subnetworks across species to identify network components in the malaria parasite *P. falciparum*.

**Results:**

Instead of only relying on sequence similarities to detect functional orthologs, our approach measures the conservation between the neighborhood subnetworks in protein-protein interaction (PPI) networks in two species, *P. falciparum *and *E. coli*. 1,082 *P. falciparum *proteins were predicted as functional orthologs of known transcriptional regulators in the *E. coli *network, including general transcriptional regulators, parasite-specific transcriptional regulators in the ApiAP2 protein family, and other potential regulatory proteins. They are implicated in a variety of cellular processes involving chromatin remodeling, genome integrity, secretion, invasion, protein processing, and metabolism.

**Conclusions:**

In this proof-of-concept study, we demonstrate that a subnetwork alignment approach can reveal previously uncharacterized members of the subnetworks, which opens new opportunities to identify potential therapeutic targets and provide new insights into parasite biology, pathogenesis and virulence. This approach can be extended to other systems, especially those with poor genome annotation and a paucity of knowledge about cellular networks.

## Background

Malaria is a major threat to public health and economic development in endemic regions. About 300-500 million cases are reported, and 1-2 million people die from malaria every year. Children and pregnant women are among the hardest hit of malaria victims. Five parasite species, *P. falciparum*, *P. vivax*, *P. malariae*, *P. ovale*, and *P. knowlesi*, cause human malaria. *P. falciparum *is the most virulent and widespread one.

The continuous morbidity or mortality of malaria is largely due to the rapid development of parasite resistance to currently available drugs and the increasing insecticide resistance of mosquito vectors. It is imperative to search for new lines of antimalarial drug and vaccine targets. The complete genome sequencing of *P. falciparum *and its sibling species and strains [1-6], the subsequent transcriptomic [7-30], proteomic [31-46], metabolic [47-54], interactomic analyses [55-60] and, most recently, next-generation sequencing [61-63] efforts have set the stage for a quantum leap in our understanding of the fundamental processes of the parasite life cycle and mechanisms of drug resistance, immune evasion, and pathogenesis. However, the paradigm of -omics driven target discovery has been significantly hampered by our limited knowledge of the cellular networks associated with parasite survival, development, transmission, invasion, and pathogenesis.

We propose to circumvent this limitation using a subnetwork alignment approach. It has been shown that network alignment offers an effective means to elucidate network structure and predict protein orthologs [64-69]. Our approach extends the concept of network alignment to align subnetworks of proteins for measuring their functional relations in a network context. It is particularly useful when the genome of interest suffers from poor annotation due to low or no sequence similarity to known proteins, a significant problem for *P. falciparum*, as over 60% of the predicted open reading frames (ORFs) were annotated as "hypothetical" without functional assignment [5]. Previously, we developed a supervised learning algorithm for remote homology detection based on support vector machines (SVMs) and profile kernels [70], and predicted a group of novel proteases [71], which were implicated in networks associated with signaling, stress response, cell cycle progression, metabolism, and invasion [72]. In this study, we attempt to identify network components beyond sequence-similarity searches.

PPI network alignment algorithms are designed to match nodes in two PPI networks such that the conserved interactions between the orthologs in the networks are captured or maximized in counts. The current network alignment algorithms are either local or global approaches. Local network alignment [64-69] aims at detecting pairs of subnetwork modules with many functional orthologs. Typically, these algorithms start from conserved regions and expand the regions greedily in the two PPI networks. Global network alignment attempts to find the best consistent mapping of the proteins in the two PPI networks for maximizing the number of conserved interactions. Previous studies tackled the global network problem with Markov Random Field (MRF) [73], combinatorial graph matching by optimization [74-76], and random walk on a Kronecker product graph of two PPI networks [77].

Since *P. falciparum *shares very few orthologous proteins with other species, the conserved interactions between *P. falciparum *PPI network and the PPI network of model organisms are too few to reveal meaningful alignments. Thus, network alignment is not directly applicable to the study of *P. falciparum *PPI network. Instead of focusing on detecting alignment, we propose to measure the functional relation between *P. falciparum *proteins and the annotated proteins in another species by aligning the neighborhood subnetworks of the two proteins. The neighborhood subnetwork of a protein (called the central protein) contains the nearby neighbors reachable by the protein through a small number of hops in the PPI network. Our assumption is that the neighborhood subnetwork captures information on the functional role of the central protein. Based on this assumption, if two proteins are functional orthologs, their neighborhood subnetworks will share similar paths or other structural patterns. Our subgraph alignment approach is designed to summarize the structural similarity between neighborhood subnetworks for ortholog prediction.

As a proof-of-concept, we chose to predict the components in the transcriptional regulation network in *P. falciparum*. It was chosen because: (1) parasite employs exquisite regulatory machinery on gene expression to assure timely and accurate coordination on parasite growth, development, infection, and virulence. (2) Very little is known about the components, dynamics, and design principles of this network. New discoveries of network components could significantly fill our knowledge gaps and possibly lead to new short lists of proteins that are poorly understood and poorly annotated for functional characterization. The correspondent network used was from *Escherichia coli*. Detection of network similarities among Eukaryotes and among Prokaryotes have been demonstrated [73,78], but detection of similarities between these two groups is a more challenging problem. The ability to make comparisons across such a wide phylogenetic gap means, firstly, that evolutionarily conserved (and therefore significant) subnetworks can be detected and, secondly, that it is possible to search beyond more closely related strains. This is especially significant in cases like *P. falciparum*, where the immediate relatives reveal comparatively little about its functional subsystems.

## Results and discussion

### Module-based subnetwork alignments predicted 1,082 components in transcriptional regulation network in *P. falciparum*

It is a common belief that the malaria parasite possesses a complex and orchestrated transcriptional regulatory system [79,80]. However, only a small number of transcriptional regulators have been identified, including a conserved set of basic transcription factors [81] and those predicted based on parasite developmental microarray expression profiles and motif analysis [82-84]. A recent study by Bischoff and Vaquero [85] combining literature searches, motif finding, and transcriptomic, proteomic, and interactomic analyses expanded this list to include proteins related to chromatin functions and remodeling.

**Figure 1:**
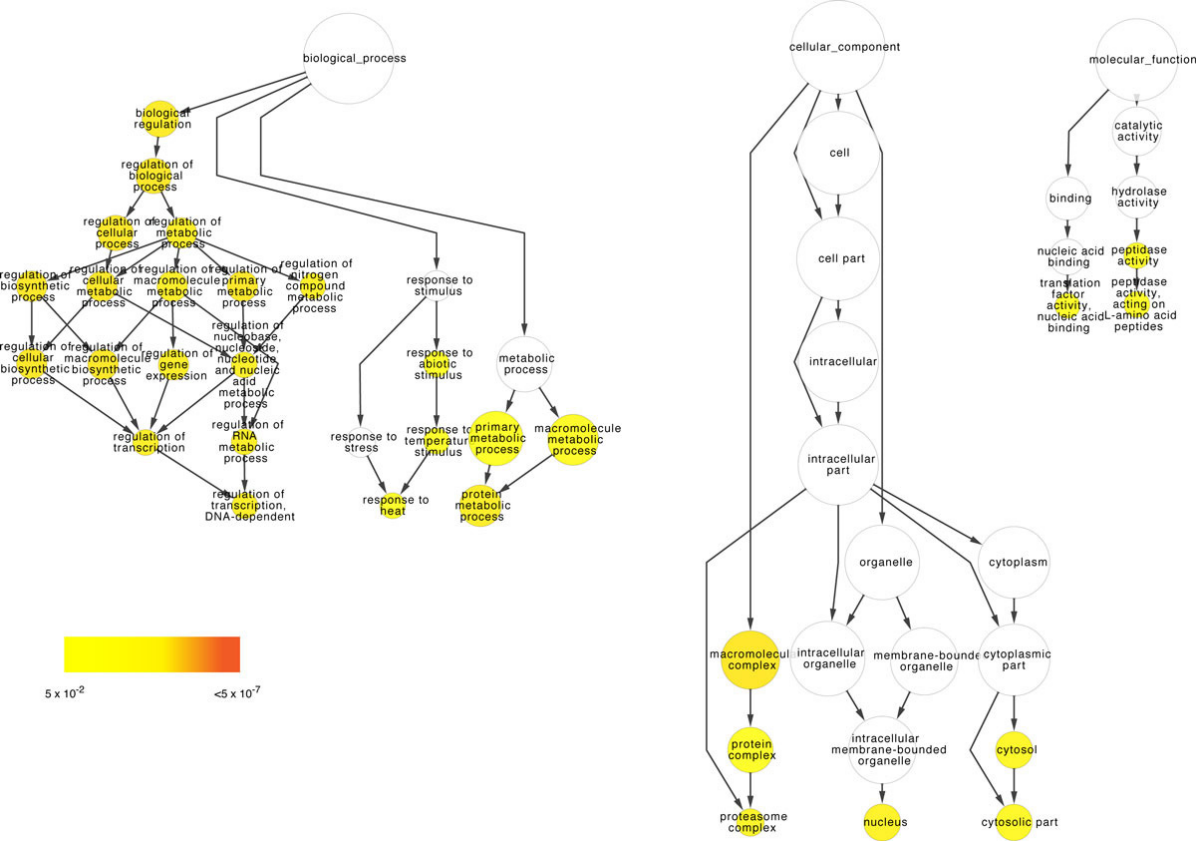
**A graphical representation of the results of a Gene Ontology analysis done using BiNGO**. The node size is proportional to the number of proteins represented by that GO term. The color represents the P-value for each enriched GO term as shown in the scale; white nodes are not enriched. The nodes are positioned to approximate their level in the Gene Ontology.

Our functional module-based subnetwork alignments predicted that 1,082 *P. falciparum *proteins were functional orthologs of known transcriptional regulators in the *E. coli *network (Additional file 1). 37% of these predicted functional orthologs appeared to be "putative uncharacterized proteins" or "conserved *Plasmodium *proteins" of unknown function. This is in agreement with the fact that 10 years after the completion of genome sequencing, the proportion of ORFs with no functional assignment has only been reduced to 45% [86]. Functional enrichment analysis [87] revealed that 31 Gene Ontology (GO) terms were over-represented (p < 0.05), including those processes that are well known to be associated with transcriptional regulation such as proteolysis [72], response to stimulus, and proteasome activity (Figure 1).

### General transcriptional regulators

**Figure 2:**
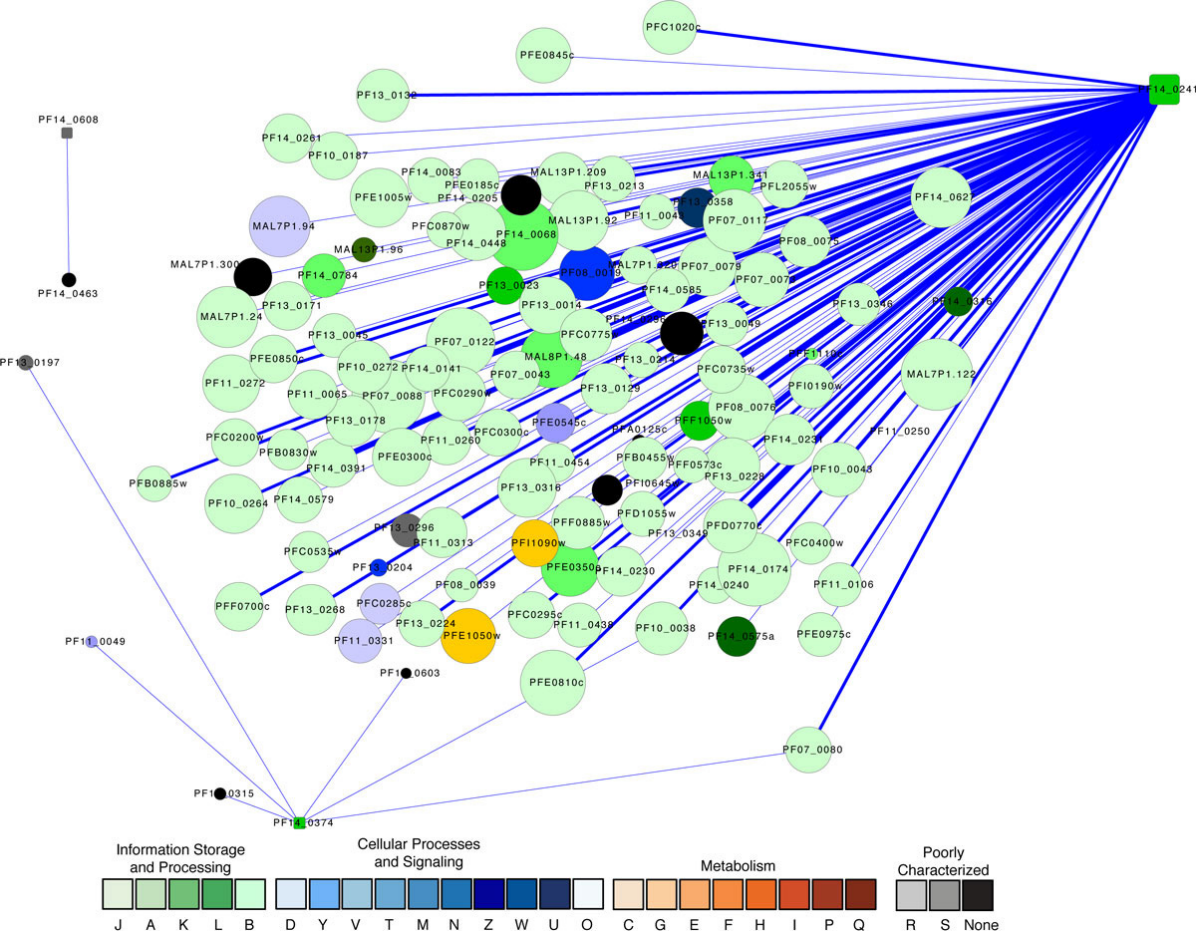
**A graph showing the proteins associated with three general transcriptional regulators**. Square nodes represent the three transcriptional regulators. Node size is proportional to the degree of the node. Nodes are colored according to their functional classification in the eggNOG database [121]. The COG categories are [122] (J) Translation, ribosomal structure and biogenesis, (A) RNA processing and modification, (K) Transcription, (L) Replication, recombination and repair, (B) Chromatin structure and dynamics, (D) Cell cycle control, cell division, chromosome partitioning, (Y) Nuclear structure, (V) Defense mechanisms, (T) Signal transduction mechanisms, (M) Cell wall/membrane/envelope biogenesis, (N) Cell motility, (Z) Cytoskeleton, (W) Extracellular structures, (U) Intracellular trafficking, secretion, and vesicular transport, (O) Posttranslational modification, protein turnover, chaperones, (C) Energy production and conversion, (G) Carbohydrate transport and metabolism, (E) Amino acid transport and metabolism, (F) Nucleotide transport and metabolism, (H) Coenzyme transport and metabolism, (I) Lipid transport and metabolism, (P) Inorganic ion transport and metabolism, (Q) Secondary metabolites biosynthesis, transport and catabolism, (R) General function prediction only, and (S) Function unknown. Confidence scores for the interactions among the nodes (S values from STRING) were divided into three groups - low (0.150-0.399), medium (0.400-0.700) and high (0.701-0.999); the groups are represented by thin, medium and heavy lines, respectively.

The predicted functional orthologs include several general transcriptional regulators (Table 1) that are commonly present in a wide variety of species. The first is basic transcription factor 3b (Accession number PF14_0241). It was found via yeast 2-hybrid (Y2H) analysis [57] to have a direct PPI with a nascent polypeptide associated complex α chain protein (PFF1050w), the erythrocyte binding antigen-181(EBA-181, PFA0125c), and a putative coronin binding protein (PFF1110c), suggesting that it may be involved in protein folding, immune evasion, and cellular actin dynamics (Figure 2). The second is a putative CCAAT-binding transcription factor (PF14_0374). A Y2H assay [57] showed that it had PPIs with six proteins. Two of these proteins are likely involved in global transcription, including (a) a putative NOT1 protein (PF11_0049). Proteins in the NOT1 family were shown to regulate the activity of general transcription factor TFIID [88]; and (b) a conserved *Plasmodium *protein (PF14_0603) that has a functional domain RPC4 which comprises a subunit of the tRNA specific polymerase RNA Pol III. The third interacting protein is a merozoite surface protein 7 (MSP7) precursor (PF13_0197), which is a regulator of parasite growth and a surface antigen regarded as a potential vaccine target [89]. The fourth protein associated with PF14_0374 is a conserved *Plasmodium *membrane protein (PF14_0315) that contains two FYVE/PHD zinc fingers for binding to potential target molecules. The remaining two proteins associated with PF14_0374 are 40S ribosomal proteins S10 (PF07_0080) and S20e (PF10_0038), indicating the interactions between transcription and translation (Figure 2).

**Table 1:** Representative *P. falciparum *proteins that were predicted to be involved in transcriptional regulation

Functional category	PlasmoDB Accession Number	Annotation
General transcription regulators	PF14_0241	putative basic transcription factor 3b
	
	PF14_0374	putative CCAAT-binding transcription factor
	
	PF14_0608	putative YL1 nuclear protein

ApiAP2	PFL1085w	putative transcription factor with 1 AP2 domain
	
	PF11_0442	putative transcription factor with 1 AP2 domain
	
	PF14_0079	putative transcription factor with 1 AP2 domain
	
	PF11_0091	putative transcription factor with 1 AP2 domain
	
	PF14_0633	putative transcription factor with 1 AP2 domain
	
	PFD0985w	putative transcription factor with 2 AP2 domains
	
	PFL1900w	putative transcription factor with 2 AP2 domains
	
	PF07_0126	putative transcription factor with 2 AP2 domains
	
	PFE0840c	putative transcription factor with 2 AP2 domains
	
	PF11_0404	putative transcription factor with 3 AP2 domains
	
	PF10_0075	putative transcription factor with 3 AP2 domains

chromosome organization	PFD0685c	structural maintenance of chromosomes protein 3 homolog
	
	MAL13P1.96	structural maintenance of chromosomes protein 2

zinc finger proteins	PF10_0091	putative zinc finger protein, C2H2 type
	
	PFL0465c	zinc finger transcription factor (Krox1), C2H2 type
	
	MAL7P1.155	putative zinc finger, C3HC4 type
	
	PF10_0046	putative zinc finger, C3HC4 type
	
	PF10_0186	putative zinc finger C-x8-C-x5-C-x3-H type
	
	MAL7P1.68	putative zinc finger protein, DHHC type
	
	PF14_0197	putative zinc finger protein, DNL type
	
	PFD0970c	putative zinc finger protein, CW type
	
	PF10_0143	putative transcriptional activator ADA2

Others	PFE0870w	putative transcriptional regulator
	
	PF14_0170	putative NOT family protein

A putative YL1 nuclear protein (PF14_0608) was predicted to be a transcriptional regulator. It has two functional domains YL1 (Pfam accession PF05764) and YL1 C-terminal domain (PF08265), both of which are typical DNA binding domains. This protein may be related to chromatin remodeling. In addition, a Y2H assay using this protein as a bait pulled out a chloroquine resistance marker protein (PF14_0463) (Figure 2).

### Apicomplexan-specific ApiAP2 transcriptional regulators

**Figure 3:**
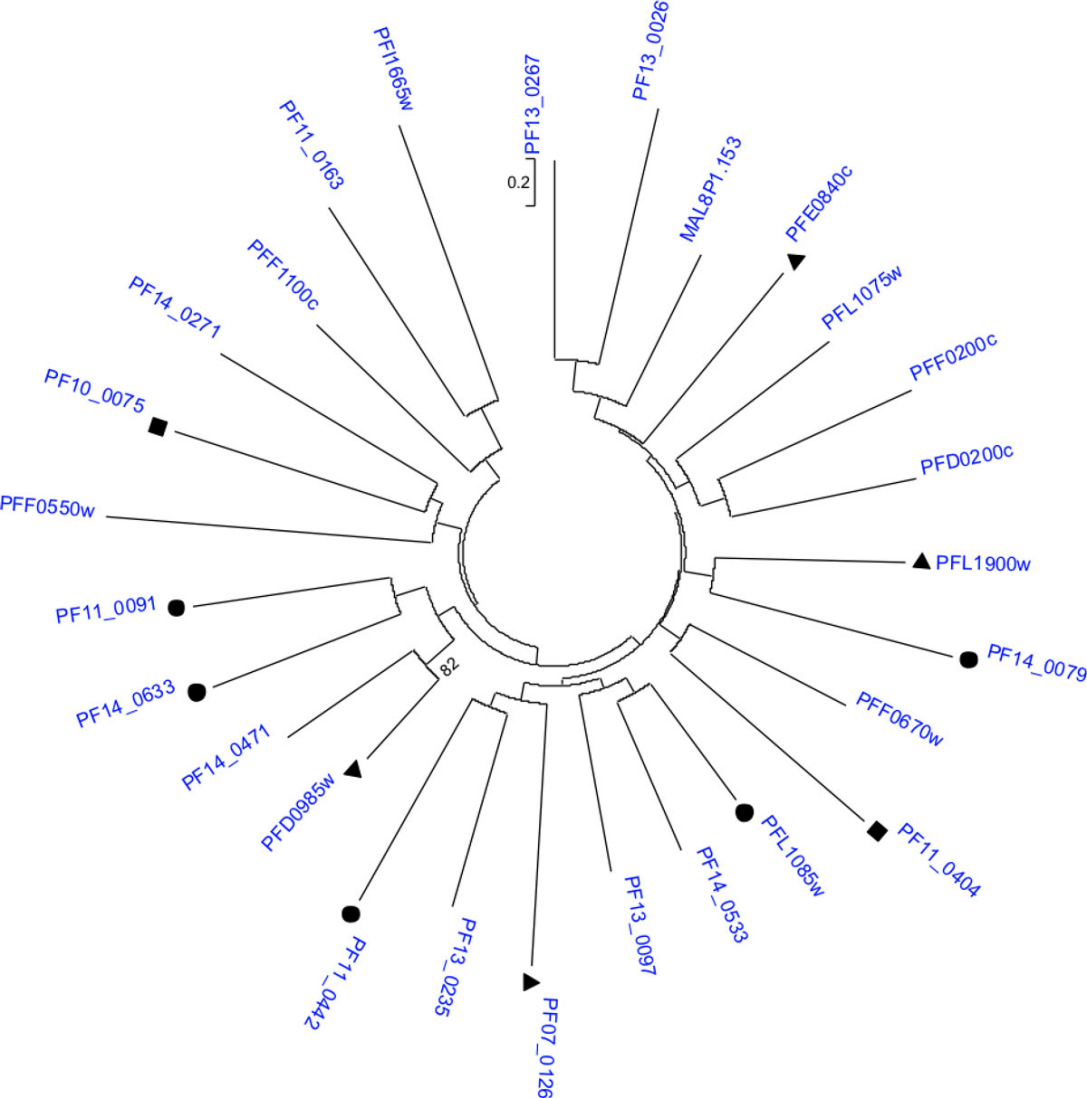
**Phylogenetic tree of the ApiAP2 transcriptional regulator family in *P. falciparum***. The tree was constructed using the neighbor-joining method [120]. 11 out of the 27 members were predicted by the subnetwork alignment algorithm. ●: ApiAP2 protein with 1 AP2 domain ▲: ApiAP2 protein with 2 AP2 domains; ■: ApiAP2 protein with 3 AP2 domains.

**Figure 4:**
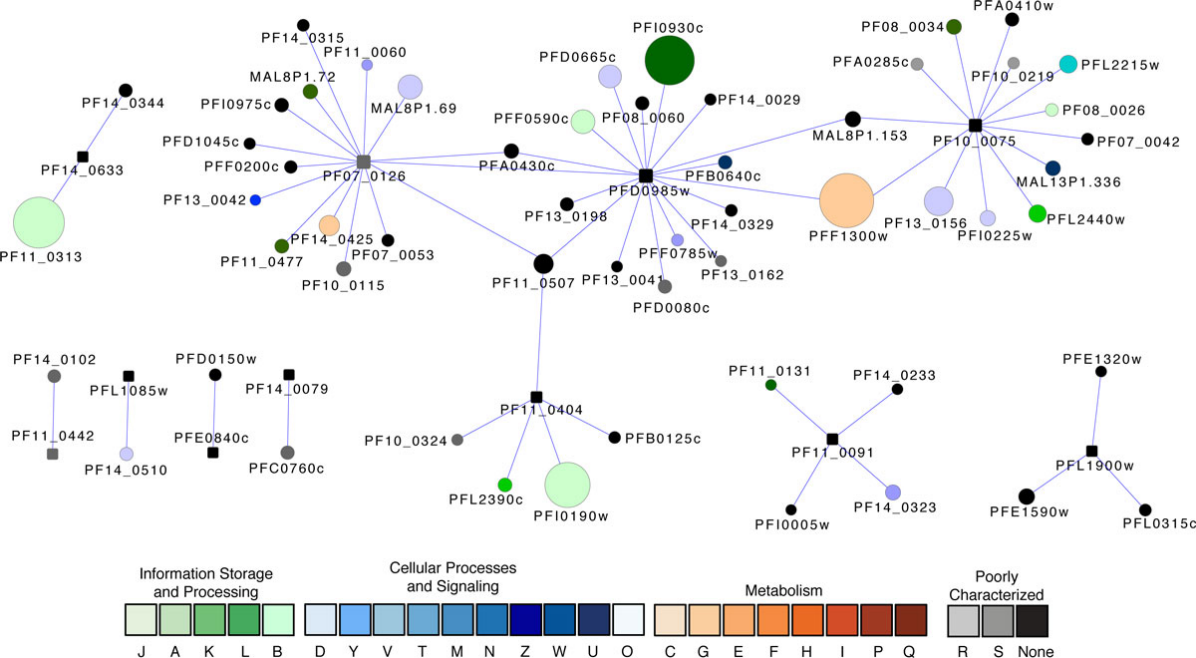
**A graph showing the proteins associated with 11 predicted ApiAP2 transcriptional regulators**. Square nodes represent ApiAP2s. Node size is proportional to the degree of the node. Nodes are colored according to their functional classification in the eggNOG database [121]. The visualization is as for Figure 2.

Most interestingly, our subnetwork alignments also predicted 11 putative transcriptional regulators belonging to the Apicomplexan-specific ApiAP2 family. A characteristic feature of this family is the presence of the Apetala2 (AP2) domain. AP2 transcription factors play a pivotal role in floral development in plants [90]. The recent discovery of AP2 in the *Apicomplexa*, the phylum to which malaria parasites belong, suggested that the ApiAP2 proteins were derived from bacteria or the apicoplast progenitor via transponsons, followed by lineage-specific radiation [91]. These ApiAP2 proteins, in addition to regulating heterochromatin formation and genome integrity, may develop novel parasite-specific functions such as antigenic variation, invasion, and sporozoite development [92-95]. *P. falciparum *possesses 27 ApiAP2 members. Among the 11 ApiAP2 proteins predicted by our network alignments, five contain a single AP2 domain, four contain two AP2 domains, and two contain three AP2 domains (Figure 3). Analyzing the protein-protein association data from the STRING database [4], in conjunction with the data from the Y2H assays, temporal microarray experiments, proteomics, and literature, revealed that these 11 ApiAP2 proteins are associated with 1-17 proteins in the cellular networks (Figure 4 and Additional File 2). At least four ApiAP2 proteins (PF07_0126, PFD0985w, PF11_0404 and PF10_0075) have PPIs, suggesting that they play central role in transcriptional regulation.

The ApiAP2 protein with highest connectivity is PFD0985w, which has 17 interaction partners (Figure 4). It has direct physical interactions with two other ApiAP2 proteins (PF07_0126 and MAL8P1.153). It is associated with a nucleosome assembly protein (PFI0930c) that is implicated in chromatin remodeling, and a putative Ndc80 homolog (PFF0785w) that may be a component of the mitotic spindle related to chromosome segregation. It is also associated with three surface antigens including a reticulocyte binding protein 2 homologue a (PF13_0198) which may play a role in determining host-cell invasion specificity [96], an antigen 332 (PF11_0506) in the Duffy binding-like (DBL) protein family which may be related to parasite entry to the host, and an asparagine-rich antigen (PF08_0060). This ApiAP2 protein PFD0985w also appeared to be related to a number of secreted proteins including a putative secreted ookinete protein (PFA0430c), and two proteins that are associated with Maurer's clefts [97], parasite-derived membranous structures within the host cell cytoplasm [PfSec31(PFB0640c), which is a COPII-coated vesicle component and PHISTb (PFD0080c)]. In addition, PFD0985w has direct PPIs with the 26S proteasome AAA-ATPase subunit RPT3 (PFD0665c), which is a component in ubiquitin-proteasome system for protein degradation, and pyruvate kinase (PFF1300w), an essential enzyme for glycolysis.

The ApiAP2 protein with second largest connectivity is PF07_0126. It has 15 PPI partners (Figure 4) that can be divided into five categories: (1) transcriptional regulation. It is associated with two otherApiAP2 proteins (PFD0985w and PFF0200c), and a CCAAT-box DNA binding protein subunit B (PF11_0477); (2) epigenetic regulation. It is associated with PfHMGB2 (MAL8P1.72), which has a DNA-binding domain: HMG-box (High Mobility Group box). The proteins in this family have been implicated in regulation of transcription, replication, repair, and chromatin remodeling; (3) signaling. PF07_0126 is associated with at least three putative signaling proteins, including (a) PF13_0042, which contains a forkhead-associated domain that is found in a variety of regulatory proteins involved in signaling. (b) a calcium/calmodulin-dependent protein kinase (PF11_0060) that is implicated in signaling cascades. (c) a putative 14-3-3 protein (MAL8P1.69). Proteins in 14-3-3 family include regulatory ligands to various signaling molecules such as kinases and receptors; (4) surface antigens for cell adhesion and entry to the host. PF07_0126 is associated with a Duffy binding-like antigen 332 (PF11_0506), an erythrocyte membrane-associated antigen (PFD1045c), and a QF122 antigen (PF10_0115) with an RNA-binding motif; (5) metabolism. The glycolytic enzyme fructose-bisphosphate aldolase (PF14_0425) is associated with the ApiAP2 protein PF07_0126.

The role of ApiAP2 proteins in transcriptional and epigenetic regulation is also indicated by a direct PPI between a putative ApiAP2 with 3 AP2 domains PF10_0075 and a histone acetyltransferase GCN5 (PF08_0034), an enzyme for histone modification and chromatin remodeling [98]. This ApiAP2 protein may also been involved in the regulation of genome integrity through a PPI with a DNA repair protein rhp16 (PFL2440w), and cytoskeleton organization of actin (Figure 4).

Two of these 11 ApiAP2 proteins have been experimentally characterized to some extent: (1) the crystal structure of the AP2 domain of PF14_0633 has been determined, revealing a multiple-site binding pattern [99], and gene disruption assays showed that its ortholog in the rodent parasite *P. berghei *was an indispensible regulator for sporozoite development in the mosquito stage [94]. However, its regulatory roles and targets remain uncharacterized in *P. falciparum*. As shown in Figure 4, it has only two direct PPIs revealed by Y2H assays [57]: the first is a ribosomal protein P0, and the second protein PTEX150 (PF14_0344) is an important component in a translocon of exported proteins (PTEX). Located in the vacuole membrane, PTEX was recently discovered as a novel ATP-dependent protein trafficking machinery [100]. Notably, PTEX150 is only present in the *Plasmodium *genus. The PPI between PTEX150 and ApiAP2 suggests that this export machinery may have parasite-specific regulation. PTEX is becoming an attractive therapeutic target due to its importance to virulence and parasite survival and its distant evolutionary relatedness to the human host. (2) PF11_0442. Its counterpart in *P. berghei *is a transcription factor that regulates ookinete-specific gene expression for parasite invasion of the mosquito midgut. PF11_0442, however, may play a role in the red blood cell (RBC) stage: It has one PPI partner, rhoptry-associated protein 1 (RAP1, PF14_0102). RAP1 is an escort protein required to localize RAP2 to the rhoptries, apical organelles essential for RBC invasion [101].

In summary, ApiAP2 proteins are a family of stage-specific transcriptional regulators for diverse processes ranging from epigenetic modification, chromosome organization and dynamics, invasion, protein sorting and trafficking, protein turnover, and metabolism.

### Other potentially important proteins that may be involved in transcriptional regulation

Module based subnetwork alignments predicted additional proteins that are likely involved in transcriptional regulation (Table 1). Two proteins (PFD0685c and MAL13P1.96) are members of the SMC (structural maintenance of chromosomes) superfamily; they both have a RecF/RecN/SMC N terminal domain and may be involved in chromatin cohesion and dynamics. A numbers of zinc-finger proteins were identified by network alignments as well. They exhibit different types of domain configurations, including the classical DNA-binding motif C2H2 found in transcription factors, the C3HC4 type (RING finger) typically found in proteins mediating ubiquitination, the C-x8-C-x5-C-x3-H (CCCH) type implicated in cell cycle regulation, the DHHC type found in proteins important for membrane association and trafficking, the DNL type implicated in protein translocation into mitochondria, and the CW type related to DNA-binding and protein-protein interaction. A putative transcriptional coactivator (ADA2, PF10_0143) has a ZZ-type zinc finger domain. ADA2 was shown, in baker's yeast and *Arabidopsis thaliana*, to physically interact with GCN5, a histone acetyltransferase and a potent transcriptional activator [102,103]. The Y2H assay in *P. falciparum *[57] revealed that ADA2 has direct physical interactions with proteins including a minichromosome maintenance (MCM) complex subunit (PF14_0177), a pre-mRNA splicing factor (PFD0265w), a heat shock protein hsp70 interacting protein (PFE1370w), a sodium-dependent phosphate transporter (MAL13P1.206), a serine/threonine protein kinase in the FIKK family (PFA0130c), cathepsin C (PF11_0174), and a mature parasite-infected erythrocyte surface antigen (PFE0040c), suggesting its potential versatile roles in DNA replication, splicing, transport, protein processing, signal transduction, and invasion.

Other putative transcriptional regulators include PFE0870 and PF14_0170. PFE0870 contains two functional domains: a FACT complex subunit (SPT16/CDC68) domain which was reported to facilitate transcriptional initiation and interact with nucleosomes and histones [104], and a histone chaperone Rttp106-like domain. This protein may be involved in heterochromatin silencing and epigenetic regulation. PF14_0170 is a putative protein in the NOT global transcriptional regulator family. Y2H assays showed that it had direct physical interactions with CCAAT-box DNA binding protein subunit B (PF11_0477), DNA topoisomerase II (PF14_0316), and calcium dependent protein kinase 1 (PFB0815w), emphasizing its involvement in general transcriptional control and chromosome topology and signaling processes. It also has a PPI with a Pf11-1 protein (PF10_0374), which may play a role in protein trafficking processes associated with Maurer's cleft.

## Conclusions

A functional-module based alignment approach was used to predict system components in transcriptional regulatory networks in malaria parasite *P. falciparum*. Our results predicted general transcriptional regulators that may regulate gene expression in a global or pleiotropic mode. Our results also imply a group of parasite-specific transcriptional regulators in the ApiAP2 family that play roles in diverse cellular processes ranging from chromatin remodeling, protein sorting and secretion, signal transduction, and invasion. Finally, our analysis has identified other potentially important proteins involved in transcriptional regulation. Our present knowledge of the transcriptional machinery and its regulatory capacity is rudimentary. The identification of network components in this machinery will open new avenues to the development of novel therapeutic targets and provide new insights into parasite biology, pathogenesis and virulence.

The premise of our subnetwork alignment approach is that functional annotations of the proteins can be transferred across species through conserved interactions in the aligned PPI networks. Under this framework, *a priori *information as to the identity or function of a gene is not necessary for the gene to be placed in a network. Thus genes identified only because of their key role in a network become potential targets. Furthermore, placement of the gene product in a systems context could, in itself, serve to identify the function of the gene product. If successfully applied, a systems biology approach circumvents the limiting factor in comparative genomics - the difficulty in obtaining reliable functional assignments.

## Methods

### Ortholog prediction by subnetwork querying

To predict functional orthologs for *P. falciparum *proteins, we formulated the problem as subnetwork querying. We first mapped the annotated *E. coli *transcriptional factors (GO:0003700: transcription factor activity) into the *E. coli *protein-protein interaction network. For each transcriptional factor, nearby neighbors were selected to form its neighborhood subnetwork. Similarly, each *P. falciparum *protein was mapped into the *P. falciparum *PPI network and a neighborhood subnetwork was built to include its nearby neighbors. Since the *E. coli *network and the *P. falciparum *network differ in size and density, the nearby neighbors were selected with a rule to control the neighborhood size. Let *N_k_*(*p*) denote the set of proteins that are exactly *k *hops from the central protein *p*. The neighborhood of central protein *p *is *N*(*p*) = *N*_1_(*p*) ∪ *N*_2_(*p*) ... ∪ *N_k_*(*p*) such that |*N*(*p*)| ≤ 500. Specifically, we first included the neighboring proteins that are 1 hop from the central protein. If the size of the neighborhood was less than 500, we continued to include the proteins that were 2 hops from the central protein. We kept increasing the hop distance until the neighborhood size was larger than 500. In other words, nearby proteins were selected by their distance to the central protein and the neighborhood size was kept below 500 unless the central protein has more than 500 direct neighbors in the PPI network.

After we obtained the neighborhood subnetwork for the *E. coli *transcriptional factors and all the *P. falciparum *proteins, we aligned each *E. coli *subnetwork against all the *P. falciparum *subnetworks. The central protein of the best-aligned *P. falciparum *subnetwork was identified as the functional ortholog of the *E. coli *transcriptional factor.

### Aligning neighborhood subnetworks with graph kernel

**Figure 5:**
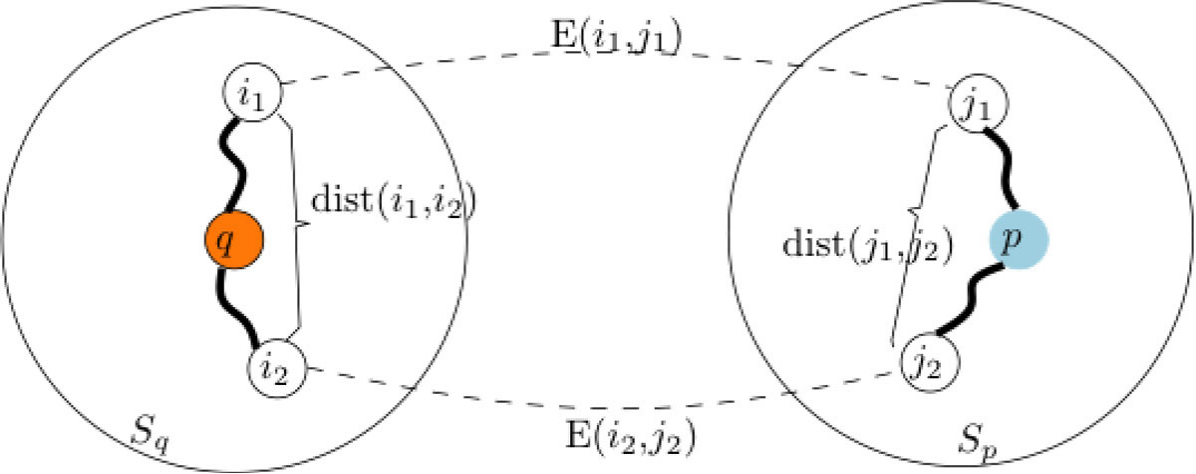
**Computation of subnetwork alignment score**. The alignment score between subnetwork *S_p _*and *S_q _*is the summation of the similarity score between all pairs of matched shortest paths ((i1, i2) and (j1, j2) in the figure), calculated based on the sequence similarities (E(i1, j1) and E(i2, j2)) and the distances in the subnetworks (dist(i1, i2) and dist(j1, j2)).

To evaluate how well a *P. falciparum *neighborhood subnetwork aligned with an *E. coli *neighborhood subnetwork, we assigned a score for each possible alignment and summarized the alignment scores with a graph kernel. Graph kernels are effective approaches to measure the similarity between two labeled networks [105,106]. Given a pair of labeled graphs, a graph kernel is designed to summarize all possible isomorphic subgraphs (exact matches) in the two graphs. However, since there are an exponential number of subgraphs, it is computationally infeasible to detect all isomorphic subgraphs. A simplification is to compute the number of common paths between two graphs by a random walk on a product graph of the two compared graphs or by dynamic programming [107-109]. Alternatively, a graph kernel can also explicitly summarize the similarity between the shortest paths in the two graphs with each pair of shortest paths measured by a convolution kernel [110]. Since our focus is only on the paths that go through the central protein, we modified the shortest path graph kernel to only consider the paths between the central protein and the other proteins in the subnetwork. The underlying hypothesis is that each shortest path going through the central protein can characterize the functional role of the protein in the chained molecular activities along the path. As shown in Figure 5, given two subnetworks *S_p _*with central protein *p *and *S_q _*with central protein *q*, we define a simple shortest path similarity function,

$$K(S_{q},S_{p})=\frac{1}{\left|S_{q}\right|+|S_{p}|}\prod_{\forall (i1,i2)\in S_{q}}B(\left(i1,i2\right),S_{p})$$

where,

$$B((i1,i2),S_{p})=\underset{\forall \left(j1,j2\right)\in S_{p}}{\max}\frac{2E\left(i1,j1\right)E\left(i2,j2\right)}{dist\left(i1,i2\right)+dist\left(j1,j2\right)}$$

$E(x,y)=exp(-\frac{Eval(x,y)}{\sigma })$ with the normalization parameter *σ *= 10 measures the sequence similarity between proteins x and y based on the E-value of the sequence alignment, and dist(x, y) is the length of the shortest path connecting proteins x and y in the PPI subnetwork. Since the scores were small numbers, the computation was done in -log_10 _scale. In this similarity function, we took each pair of the proteins (i1, i2) in one subnetwork and identify the (j1, j2) in the other subnetwork that gives the maximum ratio between their sequence similarity with respect to (*i*1, *i*2) and the closeness in the subnetworks. Specifically, we computed the shortest path through the central protein between all pairs of proteins in the neighborhood subnetwork. The shortest paths of two neighborhood subnetworks are then compared and scored pairwise. The total of the alignment scores was reported as the subnetwork alignment score. Our strategy is to incorporate both the sequence similarity of the proteins and the role of the central proteins in the subnetwork in the similarity measure, which summarizes the functional coherence between the two subnetworks and between the two central proteins of the two subnetworks.

### Network data and analysis

The *E. coli *protein-protein interactions were obtained from *IntAct *database. *IntAct *database provides binary protein-protein interactions derived from literature curation or direct user submissions. The complete set of protein-protein associations for *P. falciparum *was extracted from the STRING database [111]; each association between a pair of proteins has a confidence score (S) ranging from 0.15 to 0.999, based on the evidence from sequence similarity comparison, pathway (KEGG [112] and PlasmoCyc [52]) assignments, genome neighborhood analysis, phylogenetic inference, and literature co-occurrence. The associations were visualized in Cytoscape [113] and converted to an undirected weighted graph, where there is a single edge between any pair of proteins and the S value is used as the weight. The network was characterized using NetworkAnalyzer [114]. The default values were used for all three plugins. The set of proteins associated with transcriptional regulation were screened using BiNGO [115] to determine if any categories of proteins, as identified by their Gene Ontology terms, were enriched. The hypergeometric test was used with the Benjamini and Hochberg false discovery date correction. A significance level of 0.05 was selected.

### The omics data mining

*P. falciparum *genomic sequence and annotation data [5], transcriptomic microarray data [7,9,12], mass-spectrometry proteomic data [34,35,39,40], and protein-protein interactome [57] data for network associated proteins were downloaded from PlasmoDB (http://www.plasmodb.org) [116]. Conserved domains/motifs were identified by searching InterPro [117]. Multiple alignments were obtained using the ClustalX program [118] and T-coffee [119], followed by manual inspection and editing. Phylogenetic trees were inferred by the neighbor-joining method implemented in MEGA5 [120]. Bootstrap resampling with 1,000 replicates was carried out to assess support for individual branches. Bootstrap values of < 50% were collapsed and treated as polytomies.

## List of abbreviations used

EBA: erythrocyte binding antigen; DBL: Duffy binding-like; GO: Gene Ontology; HMG: High Mobility Group; HSP: heat shock protein; MCM: minichromosome maintenance; MSP: merozoite surface protein; MRF: Markov Random Field; ORF: open reading frame; PPI: protein-protein interaction; RAP: rhoptry-associated protein; RBC: red blood cell; SMC: structural maintenance of chromosomes; SVM: support vector machine; Y2H: yeast 2-hybrid.

## Competing interests

The authors declare that they have no competing interests.

## Authors' contributions

YW and RK conceived and designed the study. All authors performed bioinformatics data analysis and drafted the manuscript. All authors read and approved the final manuscript.

## Supplementary Material

Additional file 1 **Functional orthologs involved in transcriptional regulation in *P. falciparum***. The query genome is *P. falciparum*, and the target genome is *E. coli*. GO: Gene Ontology. BP: Biological Process. MF: Molecular Function. CC: Cellular Component. Click here for file

Additional file 2 **The protein-protein associations involving 11 ApiAP2 transcriptional regulators in *P. falciparum***. Click here for file
